# Clinicopathological Correlates of Hormone Expression-Based Subtypes of Non-Functioning Duodenal/Ampullary Neuroendocrine Tumors: A Multicenter Study of 151 Cases

**DOI:** 10.1007/s12022-025-09861-4

**Published:** 2025-05-10

**Authors:** Alessandro Vanoli, Nestor Piva, Frediano Socrate Inzani, Federica Grillo, Catherine Klersy, Silvia Uccella, Paola Spaggiari, Luca Albarello, Marco Schiavo Lena, Massimo Milione, Caterina Antoniacomi, Anna Caterina Milanetto, Alessandro Zerbi, Antonio Di Sabatino, Massimo Falconi, Andrea Anderloni, Paola Mattiolo, Claudio Luchini, Aldo Scarpa, Matteo Fassan, Paola Parente, Ombretta Luinetti, Guido Rindi, Marco Paulli, Stefano La Rosa

**Affiliations:** 1 Anatomic Pathology Unit, Department of Molecular Medicine, University of Pavia, Via Carlo Forlanini 16, 27100 Pavia, Italy; 2 Anatomic Pathology, Fondazione IRCCS San Matteo Hospital, Pavia, Italy; 3 Anatomic Pathology Department, Federal University of São Paulo, São Paulo, Brazil; 4 Anatomic Pathology, Department of Surgical Sciences and Integrated Diagnostics (DISC), University of Genova, Genova, Italy; 5 IRCCS Ospedale Policlinico San Martino, Genoa, Italy; 6 Biostatistics & Clinical Trial Center, Research Department, Fondazione IRCCS Policlinico San Matteo, Pavia, Italy; 7 Department of Biomedical Sciences, Humanitas University, Milan, Italy; 8 Department of Pathology, IRCCS Humanitas Research Hospital, Rozzano, Milan Italy; 9 Pathology Unit, IRCCS San Raffaele Scientific Institute, Milan, Italy; 10 Department of Pathology and Laboratory Medicine, First Division of Pathology, Fondazione IRCCS Istituto Nazionale Dei Tumori, Milan, Italy; 11 Pancreatic and Digestive Endocrine Surgical Research Group, Department of Surgery, Oncology and Gastroenterology, University of Padua, Padua, Italy; 12 UniCamillus - International Medical University, Rome, Italy; 13 Department of Biomedical Sciences, Humanitas University, Pieve Emanuele, Italy; 14 Pancreatic Surgery, IRCCS Humanitas Research Hospital, Rozzano, Italy; 15 Department of Internal Medicine and Medical Therapeutics, University of Pavia, Pavia, Italy; 16 First Department of Internal Medicine, San Matteo Hospital Foundation, Pavia, Italy; 17 Vita-Salute San Raffaele University, Milan, Italy; 18 Pancreatic and Transplant Surgery Unit, Pancreas Translational and Clinical Research Centre, IRCCS San Raffaele Scientific Institute, Milan, Italy; 19 Gastroenterology and Endoscopy Unit, Fondazione IRCCS Policlinico San Matteo, Pavia, Italy; 20 Department of Diagnostics and Public Health, Section of Pathology, University and Hospital Trust of Verona, Verona, Italy; 21 ARC-Net Research Centre, University of Verona, Verona, Italy; 22 Department of Medicine, Surgical Pathology & Cytopathology Unit, University of Padua, Padua, Italy; 23 Department of Pathology, Azienda ULSS2 Marca Trevigiana, Treviso, Italy; 24 Unit of Pathology, Fondazione IRCCS Ospedale Casa Sollievo Della Sofferenza, San Giovanni Rotondo, Foggia, Italy; 25 Section of Anatomic Pathology, Department of Life Sciences and Public Health, Università Cattolica del Sacro Cuore, Rome, Italy; 26 Anatomic Pathology Unit, Department of Woman and Child Health Sciences and Public Health, Fondazione Policlinico Universitario Agostino Gemelli IRCCS, Rome, Italy; 27 ENETS Center of Excellence Fondazione Policlinico Universitario A. Gemelli IRCCS-Università Cattolica del Sacro Cuore, Rome, Italy; 28 Pathology Unit, Department of Medicine and Technological Innovation, University of Insubria, Varese, Italy; 29 Pathology Unit, Department of Oncology, ASST Sette Laghi, Varese, Italy; 30 Hereditary Cancer Research Center, University of Insubria, Varese, Italy

**Keywords:** Cell subtyping, Gastrin, Null-cell tumors, Plurihormonal tumors, Somatostatin

## Abstract

Duodenal neuroendocrine tumors (Duo-NETs) may arise in the ampullary and non-ampullary duodenum. Non-functioning Duo-NETs (NF-Duo-NETs), which account for most cases, may express various hormones. Previous studies have suggested that hormone production might be associated with biological aggressiveness. Current treatment protocols are based on functionality, tumor size, and location, but small NF-Duo-NETs may also have metastatic potential. We aimed to investigate whether tumor cell subtyping, based on hormone expression, could provide further insights into NF-Duo-NET biological behavior. We analyzed the clinico-pathological correlates of hormone expression in a multicenter series of 151 NF-Duo-NETs, subdividing tumors into five subtypes: gastrin-producing G-cell NETs (Gas-NETs), somatostatin-producing D-cell NETs (Som-NETs), serotonin-producing enterochromaffin-cell NETs (Ser-NETs), plurihormonal NETs, and gastrin-, somatostatin-, and serotonin-negative NETs (GSSN-NETs). Som-NETs were the most frequent (31%), followed by plurihormonal NETs (26%), Gas-NETs (24%), GSSN-NETs (13%), and Ser-NETs (4%). Som-NETs and GSSN-NETs were more commonly located in the ampullary region and showed significantly larger size, more frequent lymphatic and/or vascular invasion, and higher pT, pN, and American Joint Committee on Cancer (AJCC-9th edition) stages compared to Gas-NETs, which were often (77%) diagnosed at AJCC stage I. Ampullary Som-NETs showed a more invasive and metastatic potential compared to non-ampullary Som-NETs, while, among plurihormonal NETs, the predominantly expressed hormone influenced tumor biological features, with gastrin-predominant NETs showing less invasive potential. At logistic regression, both tumor cell subtype and tumor size were independently associated with aggressiveness (pT3, pN1, or pM1 stage at diagnosis). Hormonal expression profiling may be clinically relevant in NF-Duo-NETs, independently of tumor size.

**Supplementary Information:**

The online version contains supplementary material available at 10.1007/s12022-025-09861-4.

## Introduction

Duodenal neuroendocrine neoplasms (Duo-NENs) are very rare, comprising only 3% of all duodenal tumors and 4–5% of all digestive NENs [1, 2], although their incidence has increased in recent decades [3].

Duo-NENs represent a heterogeneous group of neoplasms, which include epithelial neoplasms (i.e. well-differentiated neuroendocrine tumors (NETs), poorly differentiated neuroendocrine carcinomas (NECs), and mixed neuroendocrine-non-neuroendocrine neoplasms (MiNENs)), non-epithelial neoplasms (i.e., paragangliomas), and epithelial-neuronal neoplasms (i.e., composite gangliocytoma/neuroma and neuroendocrine tumors (CoGNET) [4, 5].

Duodenal neuroendocrine tumors (Duo-NETs) can arise in the non-ampullary duodenum or the regions of the major and minor papilla/ampulla [6]. These tumors may be sporadic or associated with hereditary cancer predisposition syndromes, such as multiple endocrine neoplasia type 1 (MEN1) and neurofibromatosis type 1 (NF1). Duo-NETs can be classified clinically as functioning or non-functioning tumors. Functioning tumors primarily include gastrinomas, which cause Zollinger-Ellison syndrome, serotonin-producing tumors associated with carcinoid syndrome, and NETs with ectopic hormone secretion, such as insulinomas, growth hormone-releasing hormone (GHRH)-producing NETs causing acromegaly, and adrenocorticotropic hormone (ACTH)-producing NETs causing Cushing syndrome [7].

Non-functioning Duo-NETs (NF-Duo-NETs), which account for most (> 75%) Duo-NETs, are often discovered incidentally but may also cause mass effect-related symptoms. Functioning Duo-NETs tend to exhibit more aggressive biological behavior compared to non-functioning tumors [8]. As a result, functioning tumors typically undergo surgical resection, whereas non-functioning tumors may be treated with less invasive endoscopic resection [5, 9–11]. Current treatment protocols for NF-Duo-NETs primarily consider tumor size and location [12]. Tumors larger than 10–15 mm or those located in the ampullary region generally undergo surgical resection. However, even smaller NF-Duo-NETs can develop local nodal metastasis [5, 11]. Therefore, even tumors smaller than 1 cm should be further evaluated using additional parameters such as histologic grade and clinical staging via endoscopic ultrasound (EUS) to guide therapeutic decisions [12, 13].

In other sites/organs, such as the pituitary, NETs are often classified based on their cell lineage and hormone production [14]. In the rectum and appendix, it is now recognized that there are serotonin-producing enterochromaffin (EC)-cell NETs and glucagon-related peptide-producing L-cell NETs, and that this distinction is clinically significant, as L-cell NETs generally have better prognosis than EC-cell tumors [15, 16]. NF-Duo-NETs can show immunoreactivity for various hormones, with gastrin, somatostatin, and serotonin being the three most frequently observed [7, 11, 17–21], while expression of other hormonal products, such as pancreatic polypeptide, calcitonin, insulin, and glucagon is less common. Gastrin is more frequently expressed in non-ampullary NETs, whereas somatostatin expression is more commonly seen in ampullary NETs [7, 18, 19, 21]. Although NF-Duo-NETs have generally been grouped together without consideration of their potential distinctive features related to hormone expression, somatostatin-expressing NF-Duo-NETs appear to be biologically more aggressive [5, 6].

The aim of the present study was to investigate whether tumor cell subtyping based on hormone expression can help to classify NF-Duo-NETs, providing further insights into their biological behavior.

## Materials and Method

### Study Population and Clinicopathological Characterization

A retrospective review of research databases and pathology files from the Pathology Departments of IRCCS San Matteo Hospital of Pavia/University of Pavia, University Hospital of Verona, University Hospital of Padua, ASST Sette Laghi, Varese/University of Insubria, San Martino Hospital, Genoa/University of Genoa, IRCCS Humanitas Research Hospital/Humanitas University Hospital, Milan, IRCCS San Raffaele Hospital, Milan, ‘Istituto Nazionale dei Tumori’ Hospital, Milan, and Agostino Gemelli University Hospital, Rome, was conducted to identify Duo-NENs. A total of 265 Duo-NENs (comprising 135 non-ampullary NENs and 130 ampullary NENs) diagnosed between 1980 and 2024 were retrieved. After excluding NECs, MiNENs, CoGNETs, and functioning NETs, 181 NF-Duo-NETs were selected. Thirty cases were excluded due to incomplete hormonal immunohistochemical profiles and the lack of available sections or paraffin blocks for additional immunostaining. A total of 151 NF-Duo-NETs were included in the present study, the majority of which (n = 149) have already been included in previous investigations [5, 6, 11, 22]. These cases were reinvestigated for tumor cell subtyping and updated follow-up information. Fasting plasma gastrin levels were tested in 95 patients at diagnosis and were below 150 pg/mL. As this is a multicenter retrospective study collection of laboratory data was not standardized; of note all selected cases were specifically sent for pathology analysis with no claim of functionality, neither symptoms or increased hormone plasma levels.

Clinico-pathological variables, including patient age at diagnosis, sex, presence of genetic tumor syndromes, tumor site and size, and follow-up data were obtained from diagnostic reports, clinical charts, interviews with family doctors, and/or from research databases of previous studies [5, 11]. Tumor grade (based on mitotic count and Ki67 proliferative index) according to the 2022 WHO Classification of Endocrine and Neuroendocrine Tumors [4], tumor necrosis, predominant architectural pattern (nested, trabecular, or tubular, according to the Soga and Tazawa classification) [22], presence of lymphatic and/or vascular invasion, presence of perineural invasion, extent of invasion, as well as immunohistochemical expression of pan-keratin, general neuroendocrine markers (synaptophysin and chromogranin A), gastrin, somatostatin, and serotonin, were assessed. Hormone expression was expressed as the percentage of positive tumor cells. NF-Duo-NETs were staged according to the AJCC Cancer Staging Manual, 9 th edition [23]. The technical details of immunohistochemistry varied across institutions and over time. Immunostains for gastrin, somatostatin, and serotonin, which were not included in previous investigations, were performed as part of the present study. The hematoxylin and eosin (H&E)-stained slides and immunostains of all cases were reviewed by two pathologists (AV and NP), and in the event of discrepancies, a consensus was reached.

The study was conducted in accordance with the clinical standards set out in the 1975 Declaration of Helsinki and its revision in 1983 and was approved by the Ethical Committee of Pavia (No. 20210027824).

### Tumor Cell Subtyping

NF-Duo-NETs were classified based on the immunohistochemical expression of gastrin, somatostatin, and serotonin, using previously adopted definitions [9, 24], with slight modifications to homogenize the criteria among the various groups, as follows:

i) *Gastrin-producing G-cell NETs (Gas-NETs)***:** NETs composed exclusively or predominantly (> 50% of tumor cells) of gastrin-producing cells, with other hormones expressed in minor cell populations (< 5% of tumor cells).
ii) *Somatostatin-producing D-cell NETs (Som-NETs)***:** NETs composed exclusively or predominantly (> 50% of tumor cells) of somatostatin-producing cells, with other hormones expressed in minor cell populations (< 5% of tumor cells).
iii) *Serotonin-producing EC-cell NETs (Ser-NETs)***:** NETs composed exclusively or predominantly (> 50% of tumor cells) of serotonin-producing cells, with other hormones expressed in minor cell populations (< 5% of tumor cells).
iv) *Plurihormonal NETs***:** NETs expressing at least two hormones (in any percentage of tumor cells), but not meeting the criteria for gastrin cell tumors, somatostatin cell tumors, or serotonin-producing tumors.
v) *Gastrin-, somatostatin-, and serotonin-negative NETs (GSSN-NETs)***:** NETs that lack immunoreactivity for gastrin, somatostatin, and serotonin. When additional sections were available, such GSSN-NETs were also tested for glucagon, insulin, and pancreatic polypeptide.

### Statistical Analysis

Data were described with the median and 25 th-75 th percentile if continuous and as counts and percent if categorical. They were compared between tumor cell subtypes by means of the Kruskal Wallis test and the likelihood ratio Chi squared test. Kaplan Meier survival curves were plotted, and the log rank test was used to compare survival across subtypes. The 5-year cumulative survival and 95% confidence interval (95% CI) was reported for each subtype. The median follow-up and 25 th-75 th percentile were computed with the reverse Kaplan Meier method. The independent association of tumor subtype and tumor size with an aggressive tumor, defined as a pT3, pN1 or pM1 stage at diagnosis, was assessed using a logistic model. Odds ratios (OR) and 95% CI were computed. The interaction of tumor subtype and size was tested and excluded. The model area under the ROC curve (AUC ROC) was computed for discrimination. All analyses were performed using the Stata software (release 18.5, StataCorp, College Station, TX, USA). A 2-sided p-value was considered statistically significant. For post-hoc comparisons, the Bonferroni adjusted p-value was computed to 0.0025.

## Results

The present study included 151 cases of NF-Duo-NETs. Their clinicopathological features are summarized in Supplementary Table 1. Surgical resection was performed in 70 cases, while 81 NF-Duo-NETs were removed endoscopically.

### Hormonally-Defined Tumor Subtypes

**Fig. 1:**
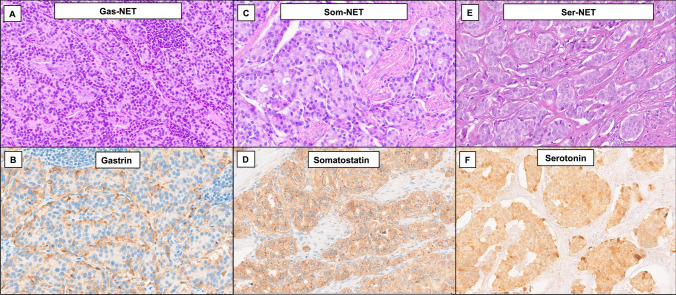
Histological images of a Gas-NET, a Som-NET, and a Ser-NET. **A-B**) Gas-NET showing a trabecular architecture and diffuse positivity for gastrin (**A**, hematoxylin–eosin; **B**, gastrin immunostaining). **C-D**) Som-NET featuring a tubular architecture and extensive positivity for somatostatin (**C**, hematoxylin–eosin; **D**, somatostatin immunostaining). **E–F**) Ser-NET exhibiting a nested structure and diffuse positivity for serotonin (**E**, hematoxylin–eosin; **F**, serotonin immunostaining)

**Fig. 2:**
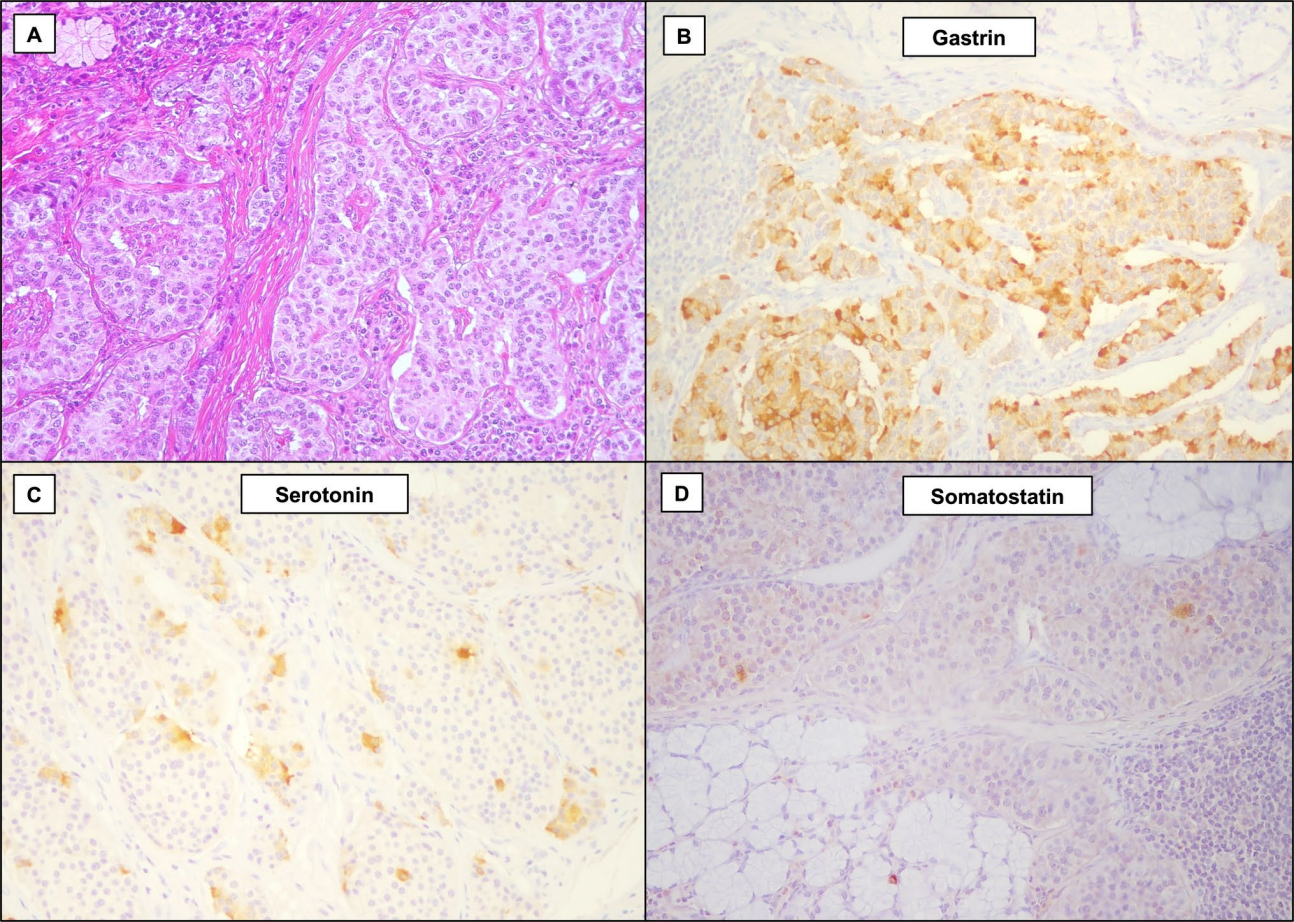
Histological images of a plurihormonal non-functioning duodenal neuroendocrine tumor. The tumor shows trabecular architecture (**A,** hematoxylin–eosin). The neoplasm exhibits expression of both gastrin (in most tumor cells; **B**, gastrin immunostaining), and serotonin (in 5–10% of tumor cells; **C**, serotonin immunostaining). Rare somatostatin-positive cells are also seen (**D**, somatostatin immunostaining)

**Fig. 3:**
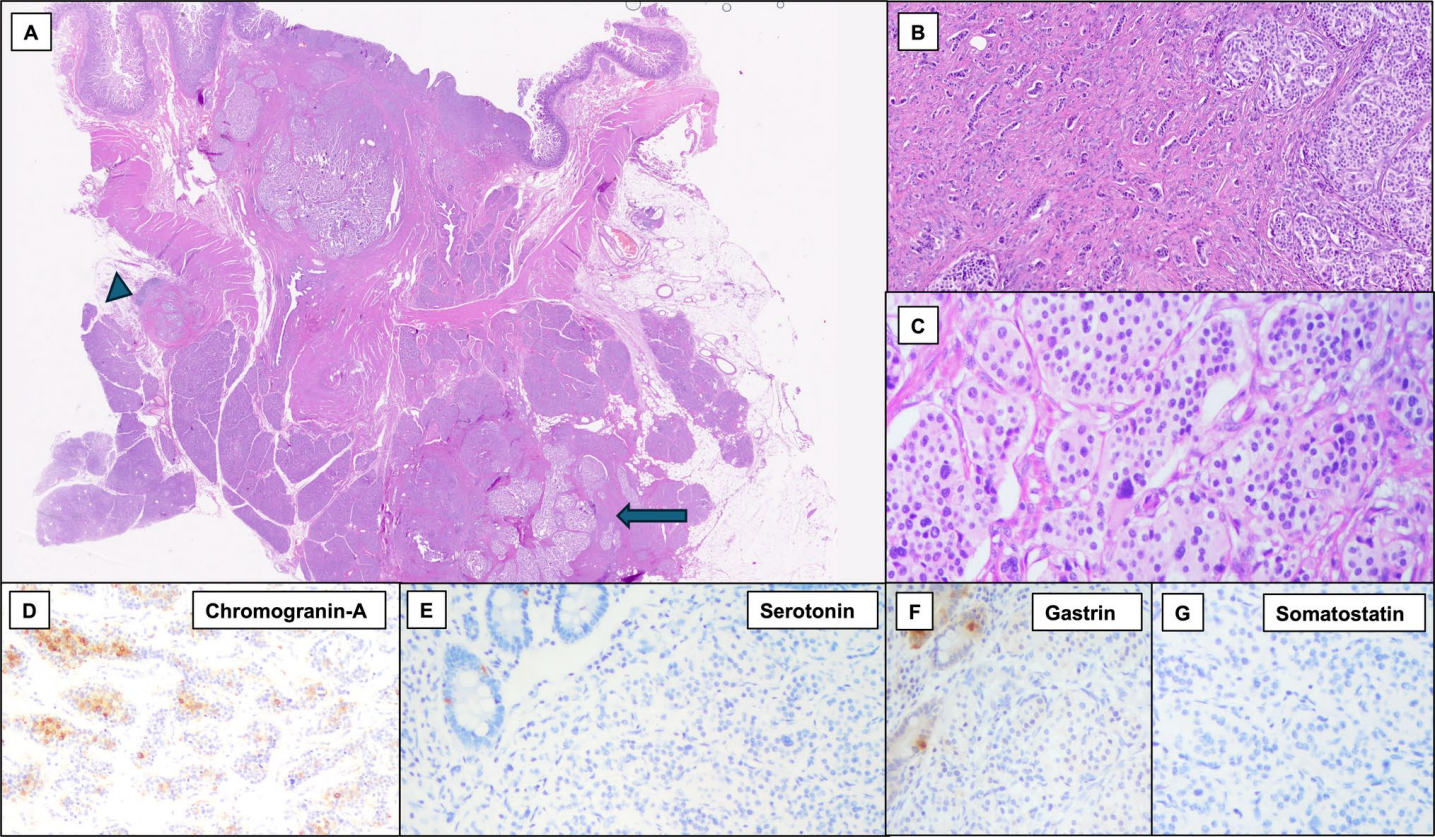
Histological images of a gastrin-, somatostatin-, and serotonin-negative neuroendocrine tumor (GSSN-NET) of the ampullary region. The low-power view (**A**) highlights a local lymph node metastasis (arrowhead) and infiltration of pancreatic parenchyma (arrow) (**A**, hematoxylin–eosin). The tumor shows a well-differentiated neuroendocrine morphology with a predominantly trabecular architecture (**B**), minor areas with a nested pattern and occasional nuclear pleomorphism (**B-C,** hematoxylin–eosin). The neoplasm shows diffuse expression of synaptophysin (not shown) and focal positivity for chromogranin A (**D**, chromogranin A immunostaining). Tumor cells are negative for serotonin (**E**, serotonin immunostaining), gastrin (**F**, gastrin immunostaining) and somatostatin (**G**, somatostatin immunostaining), with positive internal controls in the duodenal mucosa

Most NF-Duo-NETs were classified as Som-NETs (31%, Fig. 1), followed by plurihormonal NETs (26%, Fig. 2) and Gas-NETs (24%, Fig. 1). GSSN-NETs (13%, Fig. 3) and Ser-NETs (4%, Fig. 1) were rarer. Eleven out of 20 GSSN-NETs were also tested for glucagon, insulin, and pancreatic polypeptide and proved to be negative. As shown in Table 1, plurihormonal NETs most frequently expressed gastrin (82.5%), followed by somatostatin (80%). In addition, gastrin was the predominant hormone in 55% of such tumors. The most common combination was gastrin-somatostatin (40%), while combined serotonin-somatostatin expression was seen in only 7 cases (17%). Nine plurihormonal NETs (22%) were positive for all three hormones. All tumors were diffusely positive for pan-keratin and synaptophysin. Most NF-Duo-NETs (89%) diffusely expressed chromogranin A, while 16 cases, including 8 Som-NETs (17%), 3 GSSN-NETs (15%), 3 Gas-NETs (8%) and 2 plurihormonal tumors (5%), showed only focal expression of chromogranin A.

**Table 1:** Hormone expression in plurihormonal nonfunctioning duodenal neuroendocrine tumors

*Plurihormonal neuroendocrine tumors*	n = 40
***Hormone expression (in any percentage of tumor cells)***	
Gastrin expression, N (%)	33 (82.5)
Somatostatin expression, N (%)	32 (80)
Serotonin expression, N (%)	24 (60)
***Predominantly expressed hormone***	
Gastrin, N (%)	22 (55)
Somatostatin, N (%)	17 (42.5)
Serotonin, N (%)	1 (2.5)
***Hormone combinations***	
Gastrin +/somatostatin +/serotonin-, N (%)	16 (40)
Gastrin +/somatostatin-/serotonin +, N (%)	8 (20)
Gastrin-/somatostatin +/serotonin +, N (%)	7 (17.5)
Gastrin +/somatostatin +/serotonin +, N (%)	9 (22.5)

### Clinico-Pathologic Features of the Tumor Cell Subtypes

Clinicopathological data of each individual tumor cell subtype are listed in Table 2. Patients diagnosed with Som-NETs were significantly younger than those with plurihormonal tumors, with a median age at diagnosis of 58 years and 65.5 years, respectively (p = 0.001). Five out of 47 (11%) Som-NETs were associated with NF1, while 2 out of 40 (5%) plurihormonal tumors arose in patients with genetic tumor syndromes, including one somatostatin-predominant plurihormonal NET in a NF1 patient and one gastrin-predominant plurihormonal NET in a MEN1 patient. Som-NETs and Gas-NETs were slightly more frequent in males while Ser-NETs and GSSN-NETs were more common in females; however, the difference was not statistically significant. Most Gas-NETs (78%) and plurihormonal NETs (60.5%) were endoscopically resected, whereas most Som-NETs (60%), Ser-NETs (67%), and GSSN-NETs (75%) underwent surgical resection.

**Table 2:** Clinico-pathologic features of the five tumor cell subtypes of non-functioning duodenal neuroendocrine tumors

	Gas-NETs (*n* = 37)	Som-NETs (*n* = 47)	Ser-NETs (*n* = 7)	Plurihormonal NETs (*n* = 40)	GSSN-NETs (*n* = 20)	Overall *p* value	Significant post-hoc comparisons*
**Age at diagnosis, median (25 th-75 th), years**	66 (52–73)	58 (45–65)	66 (48–70)	65.5 (55–74)	61 (50–70)	0.0278	Som-NETs vs plurihormonal NETs (*p* = 0.0012)
**Patient sex**						0.244	
Female, N (%)	13 (35)	21 (45)	5 (71)	20 (50)	12 (60)		
Male, N (%)	24 (65)	26 (55)	2 (29)	20 (50)	8 (40)		
**Genetic tumor syndrome**						0.063	
Yes, N (%)	0	5 (11)	0	2 (5)	0		
No, N (%)	37 (100)	42 (89)	7 (100)	38 (95)	20 (100)		
**Tumor site**						< 0.001	Som-NETs vs Gas-NETs (*p* < 0.001); Som-NETs vs plurihormonal NETs (*p* < 0.001); GSSN-NETs vs Gas-NETs (*p* < 0.001); Plurihormonal NETs vs Gas-NETs (*p* = 0.001)
Duodenum I, N (%)	33 (89)	5 (10.5)	3 (43)	19 (47.5)	6 (30)		
Duodenum II (extra-ampullary), N (%)	0	5 (10.5)	1 (14)	4 (10)	2 (10)		
Duodenum III, N(%)	1 (3)	0	0	2 (5)	0		
Ampulla of Vater, N(%)	3 (8)	31 (66)	2 (29)	11 (27.5)	10 (50)		
Minor papilla/ampulla, N (%)	0	6 (13)	1 (14)	4 (10)	2 (10)		
**Tumor size, median (25 th-75 th), mm^**	6 (3–10)	17 (7–25)	13 (2–18)	7.50 (4–18.5)	15 (9–20)	0.0012	Som-NETs vs Gas-NETs (*p* < 0.0001); GSSN-NETs vs Gas-NETs (*p* = 0.0018)
**Tumor size^**						0.004	Som-NETs vs Gas-NETs (*p* < 0.001)
≤ 10 mm, N (%)	28 (76)	16 (35)	3 (43)	23 (57.5)	9 (47)		
> 10 mm, N (%)	9 (24)	30 (65)	4 (57)	17 (42.5)	10 (53)		
**Predominant architectural pattern (Soga type)**						< 0.001	Som-NETs vs Gas-NETs (*p* < 0.001); Som-NETs vs GSSN-NETs (*p* = 0.001); Som-NETs vs plurihormonal NETs (*p* < 0.001)
A (nested), N (%)	8 (21)	8 (17)	4 (57)	5 (12.5)	6 (30)		
B (trabecular), N (%)	28 (76)	9 (19)	2 (29)	24 (60)	11 (55)		
C (tubular), N(%)	1 (3)	30 (64)	1 (14)	11 (27.5)	3 (15)		
**Tumor grade**						0.438	
G1, N (%)	31 (84)	37 (79)	5 (71)	34 (85)	13 (65)		
G2, N(%)	6 (16)	10 (21)	2 (29)	6 (15)	7 (35)		
**Lymphatic and/or vascular invasion**						< 0.001	Som-NETs vs Gas-NETs (*p* < 0.001); GSSN-NETs vs Gas-NETs (*p* < 0.001)
Yes, N (%)	4 (11)	29 (62)	3 (43)	14 (35)	13 (65)		
No, N (%)	33 (89)	18 (38)	4 (57)	26 (65)	7 (35)		
**Perineural invasion**						0.187	
Yes, N (%)	1 (3)	8 (17)	1 (3)	5 (12.5)	4 (20)		
No, N (%)	36 (97)	39 (83)	6 (97)	35 (87.5)	16 (80)		
**Invasion beyond the submucosa**						0.006	Som-NETs vs Gas-NETs (*p* = 0.001)
Yes, N (%)	7 (19)	26 (55)	4 (57)	10 (25)	12 (60)		
No, N (%)	28 (76)	20 (43)	3 (43)	29 (72.5)	7 (35)		
Undetermined, N (%)	2 (5)	1 (2)	0	1 (2.5)	1 (5)		
**pT stage †**						0.001	Som-NETs vs Gas-NETs (*p* < 0.001); GSSN-NETs vs Gas-NETs (*p* = 0.002)
pT1, N (%)	25 (71)	12 (26)	3 (43)	23 (59)	5 (26)		
pT2, N (%)	8 (23)	17 (37)	1 (14)	10 (26)	7 (37)		
pT3, N (%)	2 (6)	17 (37)	3 (43)	6 (15)	7 (37)		
**pN stage**						0.001	Som-NETs vs Gas-NETs (*p* = 0.001); GSSN-NETs vs Gas-NETs (*p* < 0.001)
pNx, N (%)	29 (78)	19 (40)	3 (43)	25 (60.5)	5 (25)		
pN0, N (%)	2 (6)	5 (11)	2 (28.5)	6 (15)	5 (25)		
pN1, N (%)	6 (16)	23 (49)	2 (28.5)	9 (22.5)	10 (50)		
**Distant metastasis**						0.163	
Yes, N (%)	2 (5)	4 (9)	0	3 (7.5)	5 (25)		
No, N (%)	35 (95)	43 (91)	7 (100)	37 (92.5)	15 (75)		
**AJCC stage, 9 th edition ◊**						0.003	Som-NETS vs Gas-NETs (p < 0.001); GSSN-NETs vs Gas-NETs (p = 0.002)
Stage I, N (%)	23 (77)	12 (32)	3 (50)	21 (68)	4 (24)		
Stage II, N (%)	1 (3)	1 (3)	0	1 (3)	1 (6)		
Stage III, N (%)	4 (13)	21 (55)	3 (50)	6 (19)	7 (41)		
Stage IV, N (%)	2 (7)	4 (10)	0	3 (10)	5 (29)		

*For post-hoc comparisons, adjusted p-value for significance was 0.0025 (Bonferroni correction). AJCC: American Joint Committee on Cancer; Gas-NETs: gastrin-producing G cell neuroendocrine tumors; GSSN-NETs: gastrin-, somatostatin-, and serotonin-negative neuroendocrine tumors; NET: neuroendocrine tumor; Som-NETs: Ser-NETs: serotonin-producing EC cell neuroendocrine tumors; somatostatin-producing D-cell neuroendocrine tumors. †pT stage could not be assigned in 2 Gas-NETs, 1 Som-NET, 1 plurihormonal NET, and 1 GSSN-NET; ^tumor size could not be assigned in one Som-NET and one GSSN-NET; ◊AJCC stage could not be assigned in 7 Gas-NETs, 9 Som-NETs, 1 Ser-NET, 9 plurihormonal NETs, and 3 GSSN-NETs

Tumor cell subtypes showed marked differences in terms of location (p < 0.001). Gas-NETs arose almost exclusively (89%) in the first part of the duodenum, while Som-NETs (79%) and GSSN-NETs (60%) were mostly ampullary. Ser-NETs and plurihormonal tumors were more widely distributed across the duodenum.

The median size of Som-NETs (17 mm) and GSSN-NETs (15 mm) was significantly higher than Gas-NETs (6 mm) [p < 0.0001 and p = 0.0018, respectively]; the median size of plurihormonal (7.5 mm) and Ser-NETs (13 mm) was intermediate. Histologically, most (64%) Som-NETs exhibited a predominant tubular architecture, while Gas-NETs (76%), plurihormonal NETs (60%), and GSSN-NETs (55%) were mainly trabecular. Most tumors in all five subtypes were grade 1 and no grade 3 cases were identified. Although Som-NETs, Ser-NETs and GSSN-NETs showed a slightly higher proportion of grade 2 neoplasms in comparison with Gas-NETs and plurihormonal NETs, the difference was not statistically significant (Table 2). Tumor necrosis was absent in all cases.

Lymphatic and/or vascular invasion was significantly more often seen in Som-NETs (62%) and GSSN-NETs (65%) in comparison with Gas-NETs (11%) [p < 0.001]. Perineural invasion was slightly more common in Som-NETs (17%) and GSSN-NETs (20%), although the difference was not statistically significant. Invasion beyond the submucosa was more frequent in Som-NETs (55%) compared to Gas-NETs (19%) [p = 0.001]. In addition, it was also common in GSSN-NETs (60%) and Ser-NETs (57%), but rare in plurihormonal NETs (25%), without reaching statistical significance.

Som-NETs and GSSN-NETs showed significantly higher pT stage compared to Gas-NETs (p < 0.001 and p = 0.002, respectively). Specifically, 71% of Gas-NETs were pT1, compared to only 26% of both Som-NETs and GSSN-NETs. Plurihormonal NETs and Ser-NETs showed intermediate pT stage distribution. The same tendency was observed regarding local nodal metastasis (pN). Namely, lymph node metastases (pN1) were found in 50%, 49%, 28.5%, 22.5%, and 16% of GSSN-NETs, Som-NETs, Ser-NETs, plurihormonal NETs and Gas-NETs, respectively. Although distant metastasis at diagnosis was more frequent in GSSN-NETs (25%) in comparison with Som-NETs (9%), Gas-NETs (5%), plurihormonal NETs (7.5%), and Ser-NETs (0%), the differences were not statistically significant. The distribution of tumor stages (AJCC 9 th Edition) across subtypes was significantly different (p = 0.003); post-hoc comparisons showed that Som-NETs and GSSN-NETs were diagnosed at a significantly higher stage compared to Gas-NETs. Specifically, only 32% and 24% of Som-NETs and GSSN-NETs, respectively, were diagnosed at stage I compared to 77% of Gas-NETs. Specifically, 77% of Gas-NETs were diagnosed at stage I, compared to only 32% and 24% of Som-NETs and GSSN-NETs, respectively.

**Fig. 4:**
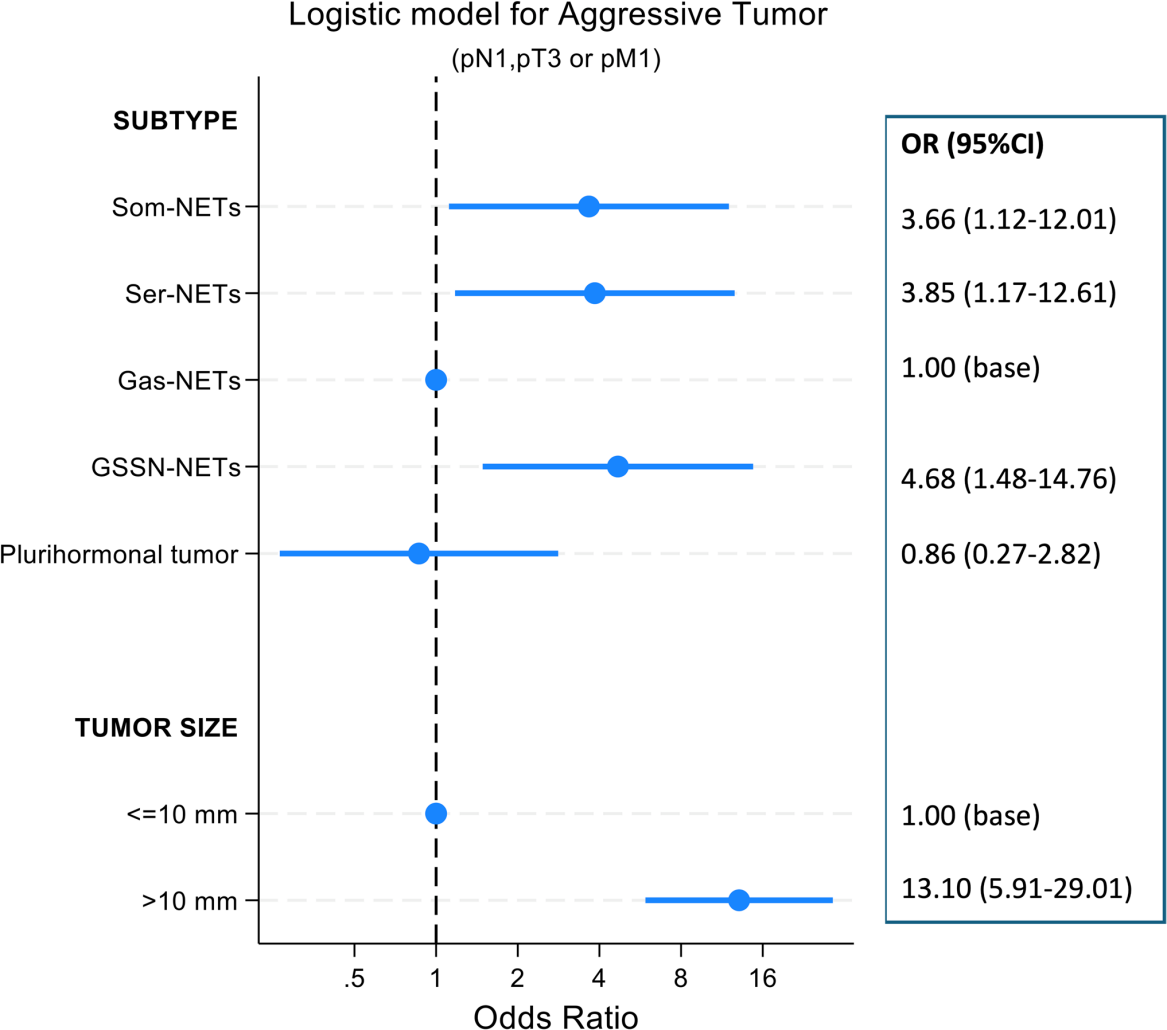
Forrest plot displaying OR (dots) and 95% CI (whiskers) estimated from a logistic model for aggressive tumor as a function of tumor cell subtype and tumor size (mm). Model Chi2 138.44, p < 0.001. Subtype: p < 0.001; Tumor size: p < 0.001. Model discrimination: AUC ROC = 0.85

At logistic regression, tumor cell subtype and tumor size were independently associated with an aggressive tumor (Fig. 4), with an increased likelihood of aggressiveness for Som-NETs, Ser-NETs and GSSN-NETs compared to Gas-NETS, together with an increased likelihood of aggressiveness for tumor size > 10 mm.

Compared to non-ampullary Som-NETs (n = 10), ampullary Som-NETs (n = 37) exhibited significantly higher proportions of cases with predominant tubular architecture, lymphatic and/or vascular invasion, grade 2, and tumor size > 10 mm (Table 3). In addition, pT2 and pT3 stages and lymph node metastases were more common in ampullary Som-NETs.

**Table 3:** Clinicopathological features of somatostatin-producing D-cell NETs (Som-NETs)

	Ampullary Som-NETs (n = 37)	Non-ampullary Som-NETs (n = 10)	p value
Age at diagnosis, median (25 th-75 th), years	57 (45–65)	58 (46–65)	0.640
Female gender, N (%)	15 (40)	6 (60)	0.273
Genetic tumor syndrome, N (%)	5 (13)*	0	0.110
Tumor size, median (25 th-75 th), mm	20 (11.5–25)	5 (3–25)	0.018
Tumor size > 10 mm, N (%)	27 (75)^	3 (30)	**0.009**
Predominant architectural pattern (Soga type)			** < 0.001**
A	4	4	
B	3	6	
C	30	0	
Tumor grade G2, N (%)	10 (27)	0	**0.019**
Lymphatic and/or vascular invasion, N (%)	27 (73)	2 (20)	**0.002**
Perineural invasion, N (%)	7 (19)	1 (10)	0.484
Invasion beyond the submucosa, N (%)	23 (62)^	3 (30)	0.125
pT stage §			**0.003**
pT1, N (%)	5 (14)	7 (70)	
pT2, N (%)	16 (44)	1 (10)	
pT3, N (%)	15 (42)	2 (20)	
pN stage			**0.008**
pNx, N (%)	11 (30)	8 (80)	
pN0, N (%)	4 (11)	1 (10)	
pN1, N (%)	22 (59)	1 (10)	
Distant metastases (pM1), N (%)	4 (11)	0	0.156
AJCC stage °			**0.002**
Stage I, N (%)	5 (17)	7 (87.5)	
Stage II, N (%)	1 (3)	0	
Stage III, N (%)	20 (67)	1 (12.5)	
Stage IV, N (%)	4 (13)	0	

*Neurofibromatosis type 1 in all cases. ^tumor size and invasion data not available in one ampullary Som-NET. §pT stage not evaluable in one ampullary Som-NET. °AJCC stage could not be assigned in 2 non-ampullary Som-NETs and in 7 ampullary Som-NETs

Among plurihormonal NETs, somatostatin-predominant NETs (n = 17) showed a significantly more frequent location in ampullary regions, a significantly higher proportion of cases with tumor size > 10 mm, predominant tubular architecture, lymphatic and/or vascular invasion, perineural invasion and invasion beyond the submucosa, as well as a higher pT stage and AJCC stage at diagnosis, compared to gastrin-predominant plurihormonal NETs (n = 22) (Table 4).

**Table 4:** Clinicopathological characteristics of plurihormonal NETs based on hormone predominance

	Gastrin-predominant plurihormonal NETs (n = 22)	Somatostatin-predominant plurihormonal NETs (n = 17)	p value
Age at diagnosis, median (25 th-75 th), years	68 (56.5–77)	61 (51–71)	0.125
Female patients, N (%)	11 (50)	9 (53)	1
Ampullary located tumors, N (%)	2 (9)	12 (71)	** < 0.001**
Tumor size > 10 mm, N (%)	4 (18)	13 (59)	** < 0.001**
Predominant C (tubular) architectural pattern	0	11 (65)	** < 0.001**
Tumor grade G2, N (%)	2 (9)	4 (23)	0.374
Lymphatic and/or vascular invasion, N (%)	2 (9)	11 (65)	** < 0.001**
Perineural invasion, N (%)	0	5 (29)	**0.011**
Invasion beyond the submucosa, N (%)*	1 (4)	9 (56)	** < 0.001**
pT stage§			**0.002**
pT1, N (%)	18 (82)	4 (25)	
pT2, N (%)	3 (14)	7 (44)	
pT3, N (%)	1 (4)	5 (31)	
pN stage			0.277
pNx, N (%)	15 (68)	9 (53)	
pN0, N (%)	4 (18)	2 (12)	
pN1, N (%)	3 (14)	6 (35)	
Distant metastases (pM1), N (%)	1 (4)	2 (12)	0.570
AJCC stage^			**0.021**
Stage I, N (%)	17 (90)	4 (36.5)	
Stage II, N (%)	0	1 (9)	
Stage III, N (%)	1 (5)	4 (36.5)	
Stage IV, N (%)	1 (5)	2 (18)	

§pT stage not evaluable in one somatostatin-predominant NET. *invasion beyond submucosa not evaluable in one somatostatin-predominant NET. ^AJCC stage not assessable in 3 gastrin-predominant NETs and in 6 somatostatin-predominant NETs

### Survival Analysis by Tumor Cell Subtype

**Fig. 5:**
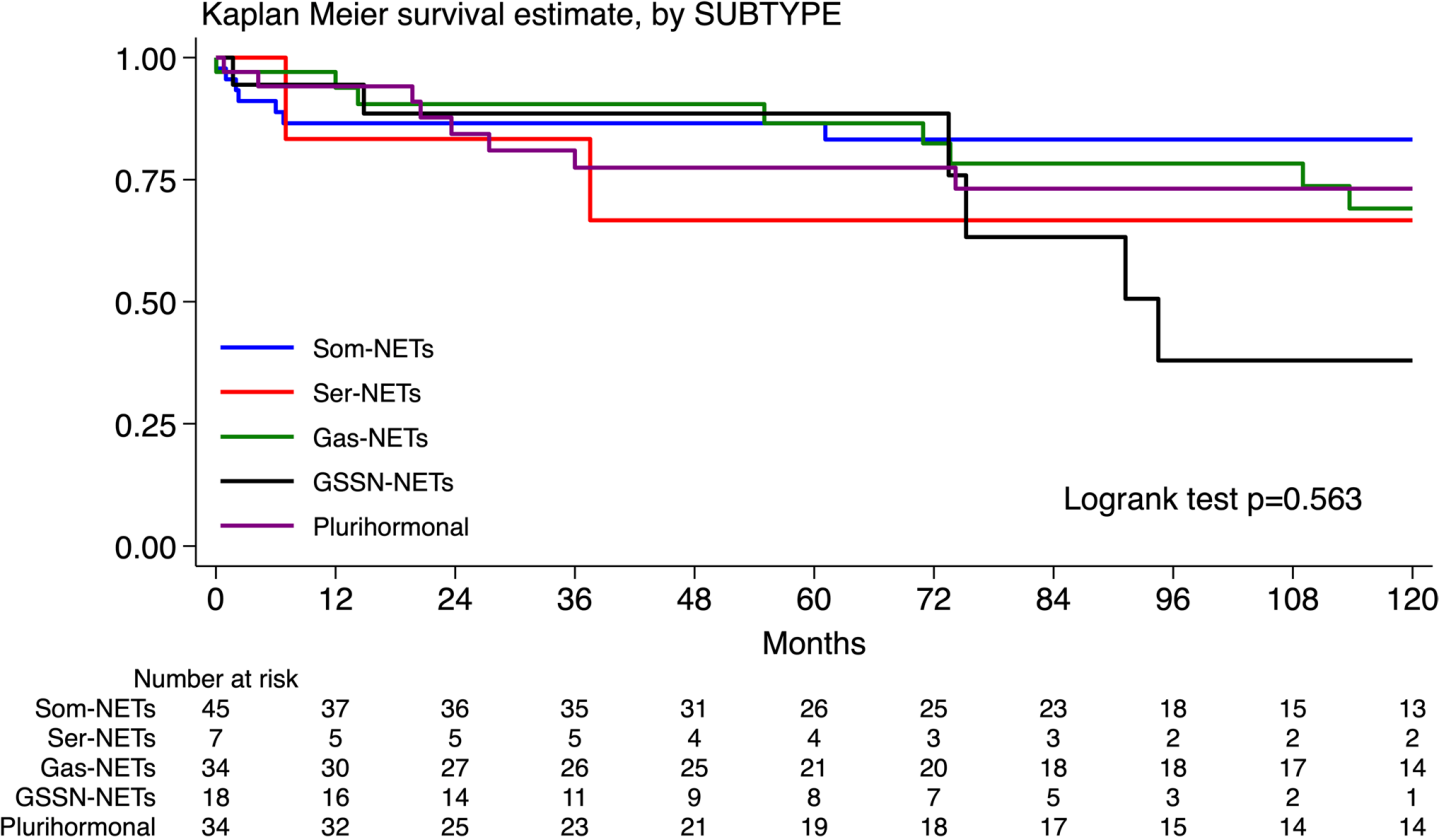
Kaplan–Meier survival estimates of patients with NF-Duo-NETs by tumor cell subtype

Thirteen patients were lost to follow-up while the median follow-up time of the whole series was 106 months (25 th-75 th: 51–172). Although the overall survival analysis showed no statistical significance among all five groups (p = 0.563, Fig. 5), the 9-year overall survival was 38% (95% CI: 9–67) for patients with GSSN-NETs, in comparison with 83% (95% CI: 68–92), 78% (95% CI: 57–90), 73% (95% CI: 53–86), and 67% (95% CI: 19–90), for patients with Som-NETs, Gas-NETs, plurihormonal NETs and Ser-NETs, respectively.

## Discussion

Our study analyzed a large series of 151 NF-Duo-NETs based on their hormone expression. Among NF-Duo-NETs, Som-NETs are the most frequent accounting for 31% of all cases, followed by plurihormonal tumors (26%) and Gas-NETs (24%). Several significant clinico-pathological correlates of the different hormone profiles emerged. Specifically, Som-NETs and GSSN-NETs showed morphological features associated with biological aggressiveness (higher rates of lymphatic and vascular invasion and higher pT, pN, and AJCC stage). On the other hand, Gas-NETs were usually small tumors and were frequently diagnosed at stage I. Such behavioral differences did not appear to be associated with tumor grade, as in our study most NF-Duo-NETs were grade 1 and there was no difference in tumor grade distribution across subtypes.

The relatively more aggressive features of Som-NETs were also seen in other luminal GI sites, such as in the rectum, where, however, they are reported to be much rarer (2%) [25]. It is important to emphasize that duodenal Som-NETs are heterogeneous neoplasms, with ampullary Som-NETs exhibiting larger tumor size and greater invasive and metastatic potential compared to non-ampullary duodenal Som-NETs, as previously suggested by our group [5, 6]. Distinctive morphological features, such as frequent tubular architecture, and a characteristic immunoprofile (common MUC1 positivity and rare SSTR2 A expression, in addition to somatostatin expression and tumor location), help to identify ampullary Som-NETs among NF-Duo-NETs. Another peculiar feature of such Som-NETs is the association with NF1 found in about 10% of these patients [5, 6, 11]. Indeed, these tumors are the most common NETs associated with NF1. This syndromic association may contribute to the younger median age at diagnosis of patients with Som-NETs compared to those with plurihormonal NETs found in our study. Although in the literature duodenal Som-NETs are frequently called “somatostatinomas”, this is a misnomer as they are not associated with the somatostatinoma syndrome or elevated serum somatostatin levels (i.e., they should be termed “non-functioning somatostatin-producing cell tumors”) [4, 5, 24, 26].

One of our most interesting findings is the relatively high frequency (13%) of cases in which the neoplasm was negative for all three hormones tested (i.e., the GSSN-NETs). Very few data concerning clinicopathological features of GSSN-NETs are described in the literature. In our study, GSSN-NETs frequently presented as deeply invasive and/or metastatic neoplasms. Whether such tumors express other hormones not tested in our study, or they represent a more immature lineage lacking any hormonal expression remains to be further investigated. Interestingly, Heymann et al. described a tendency to a worse prognosis of seven (11%) “unclassified” duodenal NETs, which were negative for gastrin, somatostatin, and serotonin, as well as for additional four hormones (calcitonin, GHRH, ACTH and vasoactive intestinal peptide (VIP)) [20]. In the pituitary, NETs that show no evidence of adenohypophysial differentiation by immunohistochemistry for pituitary hormones and the transcription factors PIT1, SF1, and TPIT (i.e. the so-called “null cell tumors”), are considered high risk [4, 27]. The relevance of transcription factors seems to be limited in duodenal NET subtyping, as both Gas-NETs and Som-NETs may express ISL1, PDX1 and rarely, CDX2, with substantial overlap among subtypes [28]. Nevertheless, further studies including other transcription factors such as ARX, which may be expressed in duodenal NETs, are needed to understand the potential relevance of transcription factor expression in duodenal NETs [29, 30]. Interestingly, both Som-NETs and GSSN-NETs are mostly located in the ampullary region and account for most of the ampullary NETs; this finding may partially contribute to explaining their relatively more aggressive behavior of ampullary NETs compared to non-ampullary Duo-NETs found in previous studies [31–34].

Ser-NETs were very rare in the ampullary/duodenal region (4% in our series), confirming findings from previous studies [7, 18], and limiting the significance of statistical analysis regarding their biological behavior, which seems however to be more aggressive compared to Gas-NETs. Duodenal Ser-NETs show similarities to their ileal counterparts as a nested insular architecture is also seen in the duodenal site. Furthermore, a little less than half of cases showed lymphatic and/or vascular invasion, and nodal metastases were observed in half of duodenal Ser-NETs with pathologically examined lymph nodes. These findings are similar to ileal Ser-NETs, which often show such features; however, in our duodenal case series, no distant metastases were observed (ileal Ser-NETs on the other hand show distant metastases in over 50% of cases) [35].

Duodenal/ampullary plurihormonal NETs have been somewhat overlooked in previous studies. However, they account for approximately one-quarter of all NF-Duo-NETs. Although the clinicopathological features of plurihormonal NETs as a whole are intermediate between Gas-NETs, on one hand, and Som-NETs and GSSN-NETs, on the other hand, our findings indicate that gastrin-predominant plurihormonal NETs differ from somatostatin-predominant plurihormonal NETs in several pathological features, with the former being more similar to Gas-NETs and the latter resembling Som-NETs. This suggests that the predominance of one hormone over the other in plurihormonal NETs may influence their histological and biological characteristics.

Although our study did not show significant differences in overall survival among all five cell subtypes, the 9-year survival of patients with GSSN-NETs (38%) was worse compared to the other subgroups, suggesting that GSSN-NET subtype may be considered an adverse prognostic factor among NF-Duo-NETs. However, further studies are needed to draw more solid conclusions.

European Neuroendocrine Tumor Society 2023 guidelines recommend a surgical approach with lymphadenectomy for NF-Duo-NETs greater than 10–15 mm and/or located in the ampullary region and/or extending beyond the submucosa and/or tumor grade G2-G3 and/or with lymphatic and/or vascular invasion [12]. In addition to the aforementioned factors, tumor cell subtyping might contribute to therapeutic decisions in NF-Duo-NETs, independently of tumor size.

The main limitations of our study are the lack of data regarding recurrence-free survival, its retrospective nature comprising a long period of time, and the lack of data on expression of transcription factors or other hormone peptides. The relative rarity of the disease also hampers to completely account for confounding factors through multivariable analyses. However, a bivariable analysis highlighted the independent association of tumor subtype with an aggressive tumor, while accounting for tumor size.

To conclude, our findings indicate that, among NF-Duo-NETs, Som-NETs and GSSN-NETs are associated with a higher invasive and lymph node metastatic potential compared to Gas-NETs. Furthermore, among Som-NETs, the more invasive and metastatic ampullary tumors should be distinguished from non-ampullary NETs, while among plurihormonal NETs, somatostatin-predominant tumors should be differentiated from the gastrin-predominant NETs with lower invasive potential. Tumor cell subtyping may have clinical relevance in NF-Duo-NETs and could potentially aid in guiding therapeutic management.

## Supplementary Information

Below is the link to the electronic supplementary material.

Supplementary file1 (DOCX 18 KB)

## Data Availability

The data that support the findings of this study are not openly available due to reasons of sensitivity and are available from the corresponding author upon reasonable request.
