# Supplementary material for: Clinicopathological Correlates of Hormone Expression-Based Subtypes of Non-Functioning Duodenal/Ampullary Neuroendocrine Tumors: A Multicenter Study of 151 Cases
Source: Endocr Pathol. 2025 May 10;36(1):18. doi: 10.1007/s12022-025-09861-4 (PMC12065733; doi:10.1007/s12022-025-09861-4)
Supplement: Supplementary file 1 — Supplementary file1 (DOCX 18 KB) [file 12022_2025_9861_MOESM1_ESM.docx]

| **Supplementary Table 1 - Clinicopathologic features of 151 non-functioning duodenal neuroendocrine tumors (NF-Duo-NETs)** | |
| --- | --- |
| **Age at diagnosis, median (25^th^-75^th^), years** | 62 (50-70) |
| **Patient sex** |  |
| **Female, N (%)** | 71 (47) |
| **Male, N (%)** | 80 (53) |
| **Genetic tumor syndrome** |  |
| **Yes, N (%)** | 7 (5)* |
| **No, N (%)** | 144 (95) |
| **Tumor site** |  |
| **Duodenum I, N (%)** | 66 (44) |
| **Duodenum II (extra-ampullary), N (%)** | 12 (8) |
| **Duodenum III, N(%)** | 3 (2) |
| **Ampulla of Vater (N, %)** | 57 (38) |
| **Minor papilla/ampulla, N (%)** | 13 (8) |
| **Tumor size, median (25^th^-75^th^), mm^** | 10 (4-20) |
| **Tumor size^** |  |
| **≤10 mm, N (%)** | 79 (53) |
| **>10 mm, N (%)** | 70 (47) |
| **Predominant architectural pattern (Soga type)** |  |
| **A (nested), N (%)** | 31 (21) |
| **B (trabecular), N(%)** | 74 (49) |
| **C (tubular), N(%)** | 46 (30) |
| **Tumor grade** |  |
| **G1, N (%)** | 120 (79) |
| **G2, N (%)** | 31 (21) |
| **Lymphatic and/or vascular invasion** |  |
| **Yes, N (%)** | 63 (42) |
| **No, N (%)** | 88 (58) |
| **Perineural invasion** |  |
| **Yes, N (%)** | 19 (13) |
| **No, N (%)** | 132 (87) |
| **Invasion beyond the submucosa** |  |
| **Yes, N (%)** | 59 (39) |
| **No, N (%)** | 87 (58) |
| **Undetermined, N (%)** | 5 (3) |
| **pT stage^** |  |
| **pT1, N (%)** | 68 (47) |
| **pT2, N (%)** | 43 (29) |
| **pT3, N (%)** | 35 (24) |
| **pN stage** |  |
| **pNx, N (%)** | 81 (54) |
| **pN0, N (%)** | 20 (13) |
| **pN1, N (%)** | 50 (33) |
| **Distant metastasis** |  |
| **Yes, N (%)** | 14 (9) |
| **No, N (%)** | 137 (91) |
| **AJCC stage, 9^th^ edition^** |  |
| **Stage I, N (%)** | 63 (52) |
| **Stage II, N (%)** | 4 (3) |
| **Stage III, N (%)** | 41 (33) |
| **Stage IV, N (%)** | 14 (12) |

*including six patients with neurofibromatosis type 1 and one patient with multiple endocrine neoplasia type 1 syndrome. ^Tumor size, pT stage and AJCC stage could not be assigned in 2, 5, and 29 cases, respectively. AJCC: American Joint Committee on Cancer

| ◊ AJCC stage could not be assigned in 7 gas-NETs, 9 Som-NETs, 1 Ser-NET, 9 plurihormonal NETs, and 3 GSSN-NETs |
| --- |

| ◊ AJCC stage could not be assigned in 7 gas-NETs, 9 Som-NETs, 1 Ser-NET, 9 plurihormonal NETs, and 3 GSSN-NETs |
| --- |

| ◊ AJCC stage could not be assigned in 7 gas-NETs, 9 Som-NETs, 1 Ser-NET, 9 plurihormonal NETs, and 3 GSSN-NETs |
| --- |
